# Single center experience with Hypotension Prediction Index (HPI) during cytoreductive surgery with Hyperthermic Intraperitoneal Chemotherapy (HIPEC)

**DOI:** 10.1186/s44158-025-00225-2

**Published:** 2025-01-22

**Authors:** Camilla L’Acqua, Luciano Frassanito, Shigeki Kusamura, Franco Valenza

**Affiliations:** 1 Department of Anesthesia and Intensive Care, Fondazione IRCCS Istituto Nazionale Dei Tumori, Milan, Italy; 2 Department of Scienza dell’Emergenza, Anestesiologiche e della Rianimazione, IRCCS Fondazione Policlinico A. Gemelli, Rome, Italy; 3 Peritoneal Surface Malignancies Unit, Colorectal Surgery, Fondazione IRCCS Istituto Nazionale Dei Tumori, Milan, Italy

To the Editor

Intraoperative hypotension (IOH) is a common condition during non-cardiac surgery [1]. A reduction of mean arterial pressure (MAP) below 65 mmHg for at least one minute is associated with an increased risk of adverse outcomes, including acute kidney injury, myocardial injury, prolonged hospital length of stay, increased in-hospital and postoperative mortality [2].

Cytoreductive surgery with hyperthermic intraperitoneal chemotherapy (CRS + HIPEC) is considered one of the most complex, high-risk, abdominal procedures. This technique has become a treatment standard for various subsets of peritoneal surface malignancies [3]. Efficient and accurate control of arterial pressure and cardiac output is a major concern during CRS-HIPEC, requiring timely fluid and vasopressors administration [3–5].

Reduction of IOH is a challenge for the clinicians: it is crucial to intervene quickly to counterbalance hemodynamic instability and prevent serious complications. The Hypotension Prediction Index – HPI The Hypotension Prediction Index—HPI (Edwards Lifesciences, Irvine, USA) is an algorithm based on a Machine learning based analysis of arterial pressure features, developed to detect detect impending hypotension [5, 6]. HPI is a unitless number that ranges from 1 to 100, and as the number increases, the likelihood or risk of a hypotensive event (defined as a MAP < 65 mmHg for more than 1 min) occurring in the near future increases.

The HemoSphere monitor platform (Edwards Lifesciences), equipped with the Acumen sensor enabled to display HPI, shows additional predictive parameters: dP/dt_max,_ (a marker of cardiac contractility), and dynamic arterial elastance (Ea_dyn_), which is the ratio of pulse pressure variation (PPV) and stroke volume variation (SVV), helping to determine fluid responsiveness.

We report our pilot experience with HPI-based hemodynamic monitoring in a cohort of patients undergoing CRS + HIPEC. Written informed consent to publication of the data has been obtained from all the patients. The research was conducted in accordance with the Declaration of Helsinki.

Ten adult patients scheduled for CRS + HIPEC were enrolled. Standard sevoflurane plus remifentanil general anesthesia was performed, and through a thoracic (T8-T9) epidural catheter an infusion of Ropivacaine 0.375% was warranted. A radial arterial catheter was connected to the HemoSphere platform. Hemodynamic management (fluids and vasopressors) followed institutional protocol (evaluation of HPI, SVV, Ea_dyn_ and dP/dt_max_), which considers the main mechanisms of hypotension (hypovolemia, vasoplegia, and decreased contractility). Recommended potential interventions were fluids, fluids plus vasopressor, vasopressor, or inotrope. All patients were scheduled to be admitted to intensive care unit (ICU) at the end of the operation.

Complete cytoreduction was achieved in all patients, and they received combination chemotherapy with: Cisplatin + Doxorubicin, cisplatin + Mitomycin-C, Mitomycin-C. The surgery lasted between 585–780 min, and the duration of HIPEC was 60–90 min. The ICU LOS was 24 h for 19 patients, 1 patient developed acute respiratory failure and stayed in ICU for 50 days.

Data in HPI group was analyzed by Acumen Analytics Software. We calculated the time-weighted average (TWA) of hypotension during surgery [5, 6]. TWA combines the duration and severity of hypotension (MAP < 65 mm Hg for at least 1 min), corrected for the total time of surgery.

In HPI group we analyzed two groups based on histopathological diagnosis: mesothelioma group (including 4 mesothelioma) and colorectal metastasis group (including 3 metastatic adenocarcinoma of colon and 3 appendix carcinoma), Fig. 1.

**Fig. 1:**
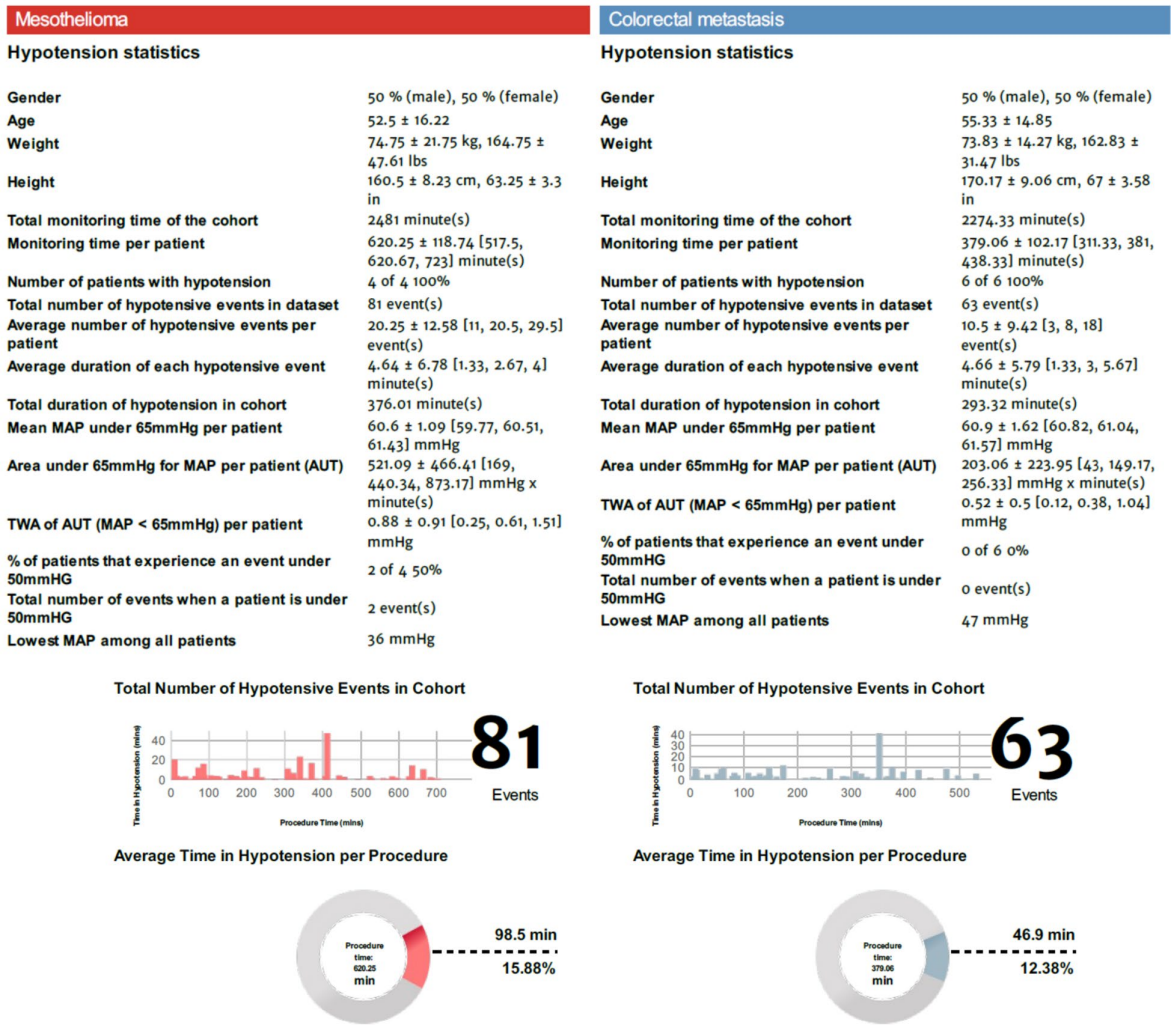
Hypotension Prediction Index description of the two groups: mesothelioma and colorectal metastasis

The median monitoring time per patient was 620.25 ± 118.74 min in the mesothelioma group and 379.06 ± 102.17 min in the colorectal metastasis group. The mean TWA-MAP < 65 mmHg per patient was 0.88 ± 0.91 mmHg in the mesothelioma group with a mean of hypotensive events per patient of 20.25 ± 12.58 and mean TWA-MAP 0.52 ± 0.5 mmHg with a mean of hypotensive events per patient of 10.5 ± 9.42 in the colorectal metastasis group. All patients experienced hypotensive events with MAP < 65 mmHg. Only in the mesothelioma group: 2 patients experienced severe hypotension with MAP < 50 mmHg (2 episodes).

The mean duration of hypotensive events per patient was similar in both group 4 ± 6 min.

According to the literature, a hypotensive burden greater than 0.3 mmHg is considered high [2, 5]. Our experience show that IOH is particularly pronounced during CRS + HIPEC. An association between hypotension and complications has been well-established [1, 3]. Our short communication does not allow to draw clinical conclusions, however, the reduction of hypotensive burden during highly impactful surgery is strongly recommended, and in our experience an early alert like HPI can be helpful.

It should be noted the difference of the oncologic status of a mesothelioma compared to a colic adenocarcinoma, with a different abdominal involvement and therefore a different hemodynamic fluctuations [7].

Unfortunately, the data related to the hypotensive burden of patients monitored without HPI were not available: further researches are required to assess the clinical benefit of HPI software during CRS + HIPEC.

In conclusion hemodynamic fluctuations and hypotensive burden during CRS + HIPEC are a major concern, especially when in mesothelioma surgery. The incorporation of HPI hemodynamic monitoring could be a valuable help to manage and reduce IOH.

Future research on hemodynamic management during major cytoreductive surgery are needed.

## Data Availability

No datasets were generated or analysed during the current study.
